# First person – Matt Johansen

**DOI:** 10.1242/dmm.049238

**Published:** 2021-09-17

## Abstract

First Person is a series of interviews with the first authors of a selection of papers published in Disease Models & Mechanisms, helping early-career researchers promote themselves alongside their papers. Matt Johansen is first author on ‘ Mycobacteriophage–antibiotic therapy promotes enhanced clearance of drug-resistant *Mycobacterium abscessus*’, published in DMM. Matt completed the research described in this article while a postdoc in the lab of Laurent Kremer at Institut de Recherche en Infectiologie de Montpellier, Montpellier, France, and is now a postdoc in the lab of Phil Hansbro at the Centre for Inflammation, Centenary Institute and University of Technology Sydney, Sydney, Australia, investigating the disease pathogenesis of infectious organisms and deciphering host–pathogen interactions.


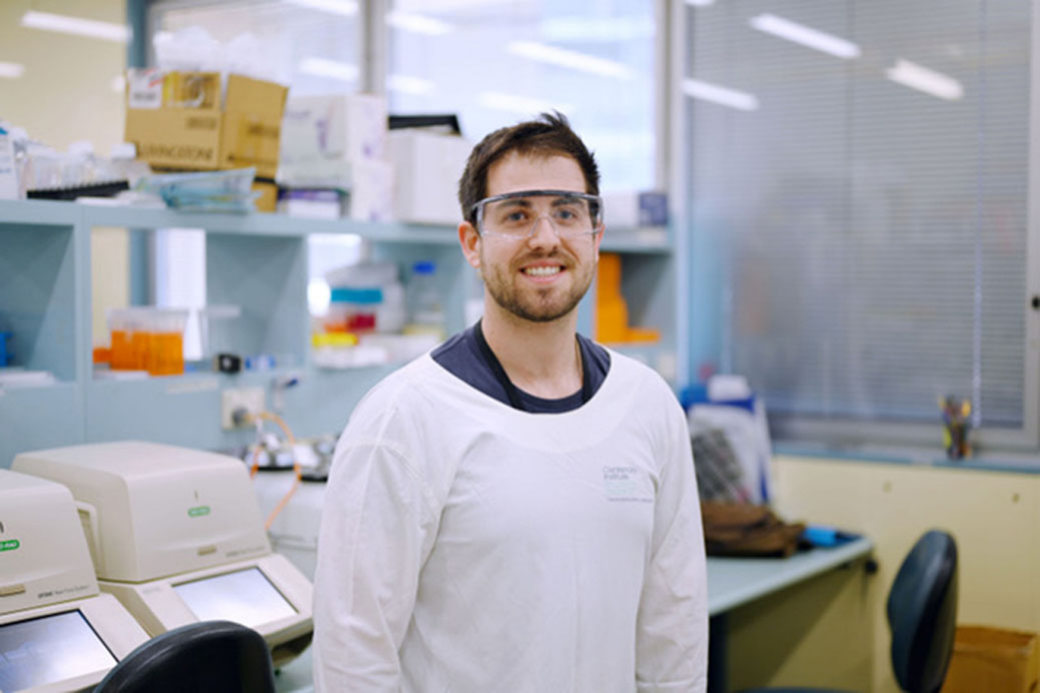


**Matt Johansen**

## How would you explain the main findings of your paper to non-scientific family and friends?

*Mycobacterium abscessus* has recently emerged as a significant pathogen causing severe lung diseases, with current treatments lacking and cure rates less than 50%. Bacteriophages are viruses that infect bacteria, and are a promising therapeutic agent for treatment of drug-resistant bacterial infections. Recently, a clinical study explored bacteriophage therapy in a cystic fibrosis patient with *M. abscessus* infection, whereby the patient was cured of infection. Based on this, we wanted to explore whether we could use the zebrafish as a model to profile bacteriophage efficacy and further improve treatment outcomes. Using this platform, we showed that bacteriophages worked remarkably well in combination with antimicrobial drugs in reducing *M. abscessus* infection in zebrafish, reducing pathophysiological signs of disease and improving survival. Thus, we can use the zebrafish model to screen novel bacteriophage prior to human clinical translation.

“The emergence of extensive antimicrobial resistance is particularly concerning and has left us with very few clinical options to treat bacterial infections in the coming years.”

## What are the potential implications of these results for your field of research?

The emergence of extensive antimicrobial resistance is particularly concerning and has left us with very few clinical options to treat bacterial infections in the coming years. These findings further strengthen the clinical implementation of bacteriophage therapy in the treatment of drug-resistant pathogens such as *M. abscessus*. Furthermore, our study showed that the combination of bacteriophages and antimicrobials further improved bacterial clearance and increased zebrafish survival, demonstrating that there is vast potential to treat drug-resistant pathogens with a bacteriophage–antimicrobial regimen. Finally, we utilised the zebrafish platform to explore bacteriophage and antimicrobial combinations, which can be used as a model system to screen novel bacteriophage and antimicrobial pairings in the future.

## What are the main advantages and drawbacks of the model system you have used as it relates to the disease you are investigating?

Zebrafish embryos are optically transparent during the early stages of life, which is a major advantage for visualising host–pathogen interactions in real time when using fluorescent bacteria and transgenic zebrafish lines, such as those with fluorescent macrophages. Furthermore, embryos possess fully functional innate immunity from as early as 30 h post-fertilisation, making them a high-throughput model system. One of the drawbacks for this model is that zebrafish do not develop adaptive immunity with functional B and T cells until a few weeks following fertilisation, by which point the embryos are more mature and have lost their transparency traits.

## What has surprised you the most while conducting your research?

I am always surprised by the creativity and ingenuity of scientists to solve real-world problems using out-of-the-box thinking and ideas. It really takes something special to come up with novel ways of solving problems that have plagued humanity for centuries, and it is a constant inspiration for me to step up and come up with my next set of new ideas for my research.

**Figure:**
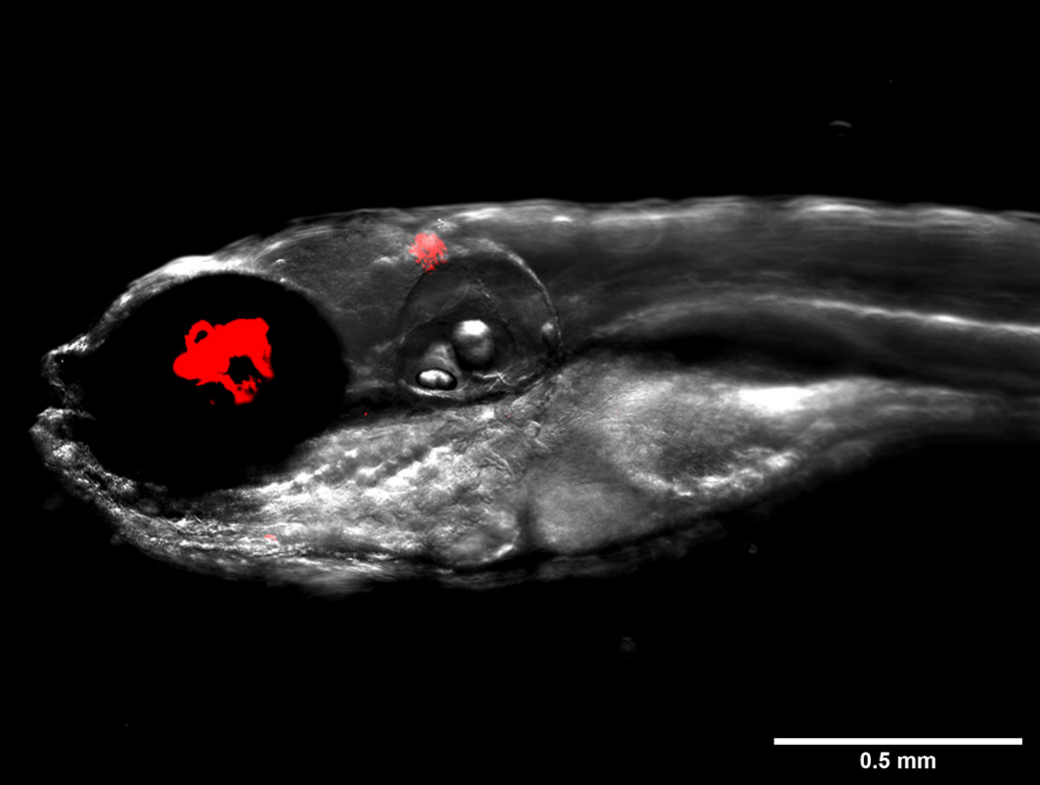
An image of a zebrafish embryo infected with *Mycobacterium abscessus* (red) depicting large serpentine cord formation.

## Describe what you think is the most significant challenge impacting your research at this time and how will this be addressed over the next 10 years?

It has become increasingly more difficult for early- and mid-career research to ‘break through’ and obtain funding and fellowship opportunities. This is particularly important as there is an ongoing need to establish and support the next generation of emerging scientific leaders. This shortage of funding is a combination of the excellent quality of early-career researchers and decreasing government funding globally. This can be addressed through raising governmental awareness on these issues and engaging commercial and philanthropic funding opportunities where possible. It is my hope that, in the next decade, we as a global scientific community can improve this situation to foster the future of scientific advancements.

“[…] providing more support and opportunities for early-career researchers in the form of grant funding or fellowships will really help deal with the issue of burn out and exit from academia […]”

## What changes do you think could improve the professional lives of early-career scientists?

As previously mentioned, I believe that providing more support and opportunities for early-career researchers in the form of grant funding or fellowships will really help deal with the issue of burn out and exit from academia that can often emerge from high-pressure environments in the early stages of any research career. It is also critical that early-career researchers are given the opportunity to individually lead and explore their own creative research interest to allow them to shape their own research careers and come up with fresh perspective on long-standing issues.

## What's next for you?

Since completing this paper, I have returned to Australia to develop the next chapter of my scientific career and establish myself as an emerging leader of microbial pathogenesis. I will continue my research into mycobacterial pathogenesis and identify new therapeutic targets and interventions to treat these highly drug-resistant pathogens. Stay tuned for the next exciting manuscript to come!
